# Enlarged pituitary gland volume: a possible state rather than trait marker of psychotic disorders

**DOI:** 10.1017/S003329172300380X

**Published:** 2024-02-15

**Authors:** Synthia Guimond, Ahmad Alftieh, Gabriel A. Devenyi, Luke Mike, M. Mallar Chakravarty, Jai L. Shah, David A. Parker, John A. Sweeney, Godfrey Pearlson, Brett A. Clementz, Carol A. Tamminga, Matcheri Keshavan

**Affiliations:** 1 Department of Psychiatry, The Royal’s Institute of Mental Health Research, University of Ottawa, Ottawa, ON, Canada; 2 Department of Psychoeducation and Psychology, Université du Québec en Outaouais, Gatineau, QC, Canada; 3 Department of Psychiatry, Massachusetts Mental Health Center and Beth Israel Deaconess Medical Center, Harvard Medical School, Boston, MA, USA; 4 Department of Psychiatry, McGill University, Montréal, QC, Canada; 5 Douglas Mental Health University Institute, Verdun, QC, Canada; 6 Department of Biomedical Engineering, McGill University Montréal, QC, Canada; 7 Cerebral Imaging Centre, Douglas Mental Health University Institute, Verdun, QC, Canada; 8 Department of Psychology, BioImaging Research Center, University of Georgia, Athens, GA, USA; 9 Department of and Neuroscience, BioImaging Research Center, University of Georgia, Athens, GA, USA; 10 Department of Human Genetics, Emory University School of Medicine, Atlanta, GA, USA; 11 Department of Psychiatry, University of Cincinnati, Cincinnati, OH, USA; 12 Department of Psychiatry, Yale University, New Haven, CT, USA; 13 Department of Neuroscience, Yale University, New Haven, CT, USA; 14 Department of Psychiatry, UT Southwestern Medical Center, Dallas, TX, USA

**Keywords:** bipolar, cognition, imaging, pituitary gland, psychosis, psychotic disorder, schizophrenia, symptom severity

## Abstract

**Background.:**

Enlarged pituitary gland volume could be a marker of psychotic disorders. However, previous studies report conflicting results. To better understand the role of the pituitary gland in psychosis, we examined a large transdiagnostic sample of individuals with psychotic disorders.

**Methods:**

The study included 751 participants (174 with schizophrenia, 114 with schizoaffective disorder, 167 with psychotic bipolar disorder, and 296 healthy controls) across six sites in the Bipolar-Schizophrenia Network on Intermediate Phenotypes consortium. Structural magnetic resonance images were obtained, and pituitary gland volumes were measured using the MAGeT brain algorithm. Linear mixed models examined between-group differences with controls and among patient subgroups based on diagnosis, as well as how pituitary volumes were associated with symptom severity, cognitive function, antipsychotic dose, and illness duration.

**Results.:**

Mean pituitary gland volume did not significantly differ between patients and controls. No significant effect of diagnosis was observed. Larger pituitary gland volume was associated with greater symptom severity (*F* = 13.61, *p* = 0.0002), lower cognitive function (*F* = 4.76, *p* = 0.03), and higher antipsychotic dose (*F* = 5.20, *p* = 0.02). Illness duration was not significantly associated with pituitary gland volume. When all variables were considered, only symptom severity significantly predicted pituitary gland volume (*F* = 7.54, *p* = 0.006).

**Conclusions.:**

Although pituitary volumes were not increased in psychotic disorders, larger size may be a marker associated with more severe symptoms in the progression of psychosis. This finding helps clarify previous inconsistent reports and highlights the need for further research into pituitary gland-related factors in individuals with psychosis.

## Introduction

Psychotic disorders are among the leading contributors of negative life outcomes and trajectories (Guilera et al., 2012). Identifying biomarkers of psychotic disorders could help predict outcomes and guide personalized treatments (Kraguljac et al., 2021). Enlarged pituitary gland is observed in the development of psychosis and could be a useful biomarker (Takahashi et al., 2013). However, it remains unclear whether enlarged pituitary gland is a state or trait marker of individuals with psychotic disorders (Aiello, Horowitz, Hepgul, Pariante, & Mondelli, 2012; Nordholm et al., 2013; Shah et al., 2015).

Previous studies investigating pituitary gland volume in psychotic disorders report mixed findings. On one hand, studies find significantly larger pituitary glands in individuals living with psychotic disorders compared to controls (Büschlen et al., 2011; Pariante et al., 2005, 2004; Takahashi et al., 2018, 2013, 2011). On the other hand, studies report significantly smaller pituitary glands, as well as non-significant differences (Gurok et al., 2019; Habets et al., 2012; Klomp, Koolschijn, Hulshoff Pol, Kahn, & Van Haren, 2012; Nicolo et al., 2010; Upadhyaya, El-Sheikh, MacMaster, Diwadkar, & Keshavan, 2007). There is an important heterogeneity in the various clinical diagnoses included in those studies which could in part explain those mixed results. There is also minimal research investigating pituitary gland volume in bipolar disorder with psychotic episodes which have yielded mixed results (Clark, Mackay, & Goodwin, 2014; Delvecchio et al., 2018; Delvecchio, Altamura, Soares, & Brambilla, 2017; Sassi et al., 2001).

In a meta-analysis, Nordholm et al. (2013) report solely a trending significant effect for a larger pituitary gland volume in ultra-high-risk individuals who transitioned to psychosis and in individuals experiencing a first episode of psychosis. No significant or trending significant differences in pituitary gland volume are found among individuals living with more enduring schizophrenia. Similarly, a more recent meta-analysis by Saunders, Mondelli, and Cullen (2019) found that high risk individuals (i.e., with schizotypal personality disorder and psychotic-experiences) show a significant increase in pituitary gland volume. However, no significant differences in pituitary gland volume were observed in clinical high-risk individuals and individuals with family history of psychosis (Saunders et al., 2019). Consistent with previous meta-analyses, the authors observed that individuals who later transitioned to developing psychosis had a significantly increased pituitary volume compared to controls. The authors suggest that pituitary volume enlargement may serve as a proxy for illness severity. Shah et al. (2015) also observed larger baseline pituitary gland volume in individuals at familial risk of psychosis who later developed psychosis. Those findings suggest a greater vulnerability of the pituitary gland function in the early stages of the illness (Klomp et al., 2012; Soni et al., 2017).

The pituitary gland is a dynamic organ and multiple factors can impact its volume over the risk period, onset, and course of the illness. Yet, whether the factors associated with psychosis are related to the volume of the pituitary gland remain to be elucidated (Borges, Gayer-Anderson, & Mondelli, 2013). Increased pituitary gland volume is positively correlated with positive schizotypal score in youth at familial risk for psychosis (Shah et al., 2015). Some data also suggest that pituitary gland volume enlargement is associated with less improvement in psychotic symptoms in individuals with early psychosis (Garner et al., 2005; Takahashi et al., 2011). Cognitive symptoms, such as impaired memory and executive functioning, are also associated with abnormal hypothalamic–pituitary–adrenal (HPA) axis functioning, and are known to be a core feature of individuals with psychotic disorders (Cullen et al., 2014; Hill et al., 2013; Kelly et al., 2019; Walder, Walker, & Lewine, 2000). Changes in pituitary gland volume during the early stages of psychotic disorders may be associated with an exacerbation of clinical and cognitive symptoms.

Furthermore, antipsychotic medication may impact pituitary gland volume. In fact, antipsychotic treatment can result in an enlargement of the pituitary gland in individuals with first-episode and enduring schizophrenia (Klomp et al., 2012; MacMaster et al., 2007; Takahashi et al., 2018). However, studies observe similar pituitary gland volume enlargement in prodromal and antipsychotic-naïve patients compared to controls, which suggests that pituitary gland volume may not be solely influenced by antipsychotic medication (Pariante et al., 2005; Takahashi et al., 2009). Also, Nicolo et al. (2010) report that when administered in a dose-dependent manner, atypical antipsychotics reduce pituitary gland volume, in contrast to previous studies. These mixed results highlight the need for further research with larger samples.

The ability to identify biomarkers for psychotic disorders has the potential to both forecast outcomes and support the implementation of tailored treatment plans (Kraguljac et al., 2021). The current study aims to determine whether individuals with psychotic disorders have larger pituitary gland volumes compared to healthy controls and to investigate the clinical associations of pituitary volumes.

To accomplish this goal, the pituitary gland volume was measured for the first time in a large and transdiagnostic group of individuals with psychotic disorders (including schizophrenia (SZ), schizoaffective disorder (SZA), and psychotic bipolar disorder (PBD)) using an automated multi-atlas segmentation pipeline. We investigated factors that may be related to the pituitary gland function and are important for psychosis, such as the severity of clinical symptoms, duration of illness, cognitive symptoms, and the level of medication intake (as measured by the chlorpromazine (CPZ) equivalent dosage).

We hypothesized that individuals on the psychosis spectrum would have larger pituitary glands than healthy controls. Furthermore, we hypothesized that an enlarged pituitary gland will be associated with greater severity of clinical symptoms, shorter duration of illness, higher level of cognitive impairments and higher dosage of antipsychotic treatments.

## Methods and materials

### Participants

Participants were recruited as part of the Bipolar-Schizophrenia Network on Intermediate Phenotypes (B-SNIP1) study, through regional advertising for research in Baltimore, Maryland; Boston, Massachusetts; Chicago, Illinois; Dallas, Texas; and Hartford, Connecticut. The participants engaged in the full clinical characterization and dense phenotyping, which included brain imaging assessments and electrophysiological assessments. Recruitment, interviews, and data collection were completed at each B-SNIP consortium site. Full details for B-SNIP1 are available in Tamminga et al. (2014). The Institutional Review Board at every participating institution approved the projects; all participants provided informed consent prior to participation after they obtained a complete study description.

After quality control of the data, a total of seven hundred and fifty-one participants were included in the study (174 individuals with SZ, 114 with SZA, 167 with PBD, and 296 healthy controls (HC). The participant demographics such as sex, age, race, as well as duration of illness, and antipsychotic dosage (CPZ equivalents) were recorded. Participants with psychotic disorders in B-SNIP1 were also classified into Biotypes, based on cognitive and electrophysiological assessments. Additional exploratory analyses using those Biotypes can be found in online Supplementary Material.

### Clinical and cognitive assessments

All assessments were typically completed within a month after enrollment.

### Clinical measures

Trained raters assessed symptom severity in patients with the Positive and Negative Syndrome Scale (PANSS) (Kay, Fiszbein, & Opler, 1987). Periodic meetings were conducted for rater training to standardize symptom ratings across all sites. Established ‘gold standard’ interviews were used during these meetings. Prior to the study, an in-person training session was held for all raters, and they were required to achieve a reliability score of over 0.85 before being allowed to administer scales. To maintain reliability, rater training was repeated every year. Duration of illness was defined as the difference between age of the participant and the age of onset. Age of onset was defined as the self-reported age of the first symptoms related to their diagnosis.

### Cognitive measures

Brief Assessment of Cognition in Schizophrenia (BACS) assesses the following cognitive domains: executive function, verbal fluency, verbal memory, working memory, attention, and motor speed. The BACS total composite score was standardized from each test with a mean score from healthy controls set to zero and the standard deviation set to one (Keefe et al., 2004).

### MRI acquisition and preprocessing

The study acquired T1-weighted scans using 3 T scanners from different manufacturers such as Achieva, Philips, GE Signa, Siemens Allegra, and Siemens Trio and sequence parameters were standardized across all sites and established from the Alzheimer’s Disease Neuroimaging Initiative protocol (6), (see online Supplementary Material). We performed quality control on a total of 911 scans, of which 63 were excluded from the study because of artifacts or motion. Preprocessing of T1-weighted images was carried out using the minc-bpipe-library (https://github.com/CobraLab/minc-bpipe-library). Quality control on all scans was performed by visual inspection. Motion artifacts were first graded (0 = no/very subtle motion, 1 = moderate motion, and 2 = severe motion), and scans with moderate-to-severe motion artifacts were excluded from the study. Freesurfer 6.0 was used to estimate the total intracranial volume (ICV) to control for its variability among participants in our analyses.

### Pituitary gland segmentation

In order to measure the sizes of the pituitary glands, we employed a label-fusion technique called the MAGeT brain algorithm to automatically segment the pituitary gland (Chakravarty et al., 2013). The MAGeT brain algorithm has been shown effective for segmenting subcortical structures such as the striatum, globus pallidus, thalamus, and the hippocampus (Chakravarty et al., 2013; Park et al., 2014). Importantly, MAGeT brain has also been previously validated to segment the pituitary gland showing high spatial overlap between manual segmentation of the pituitary gland volume and their respective automatically generated labels (Wong et al., 2014).

In the current study, a representative sample of 68 participants, matched in terms of age, sex, and diagnosis groups, had their pituitary glands manually segmented by YT using the 3D Slicer software (Egger et al., 2013). The location of the pituitary gland was identified using sagittal, coronal, and axial perspectives of the MR images. The demarcations of the pituitary gland encompassed both the anterior and posterior parts, as outlined in detail in the study by Peper et al. (2010). Following the manual segmentation, these 68 participants were inputted into the MAGeT brain pipeline as atlases, templates, and participants. Each atlas segmentation was compared to the manual delineations of all other participants using dice-kappa, and the seven manual segmentations with the highest mean dice-kappa were selected as the atlases for the final MAGeT brain pituitary gland segmentation in our sample (mean dice-kappa = 0.73). Pituitary gland volume was then automatically segmented in our whole sample, entering only those seven best manual segmentations as atlases. After rigorous quality control visual inspection, the pituitary gland was deemed successful in 751 participants, and 97 participants were excluded from the analyses due to segmentation failure. An example of the pituitary gland segmentation is provided in online Supplementary Material.

### Statistical analyses

All statistical analyses were conducted in R (version 1.3.1056) and the *lmerTest* package (version 3.1.3) was used for all linear mixed effects models (Kuznetsova, Brockhoff, & Christensen, 2017).

### Demographic and clinical data

Demographic and clinical data were assessed between each clinical diagnosis or Biotype, and healthy controls when appropriate using ANOVAs (i.e. age, duration of illness, severity of symptoms, and CPZ equivalents) and chi-square tests (i.e. sex, race, and site). Log transformations were applied to CPZ equivalent data to make it conform to normality as the data were skewed.

### Between-group comparisons

A linear mixed effects model was used to determine whether the mean pituitary gland volume was different between the group of patients with psychosis and healthy controls. Then, we performed further linear mixed effects models to investigate differences in pituitary volume between clinical diagnosis and healthy controls. All models included age, sex, race, and total ICV as covariates, as well as site and scanner as random factors. Post-hoc pairwise comparisons were also conducted.

### Factors influencing the pituitary gland volume in individuals with psychotic disorders

A series of linear mixed effects models were used to separately investigate the impact of clinical symptom severity, duration of illness, cognitive functioning and CPZ equivalents on the pituitary gland volume. Post-hoc linear mixed effects models were also performed to investigate associations between specific types of clinical symptoms and the pituitary gland volume (i.e. PANSS positive symptoms, PANSS negative symptoms, PANSS general).

To determine the strongest predictor variable for the pituitary gland volume, a final linear mixed effects model was constructed including the four factors (i.e. clinical symptom severity, duration of illness, cognitive functioning and CPZ equivalents). By considering all the factors together, we minimize the risk of Type I errors associated with multiple comparisons, as the model itself adjusts for the interactions and dependencies among the variables. We also conducted Pearson’s correlations between those factors.

All models included age, sex, race, and total ICV as covariates, as well as site and scanner as random factors. Finally, we explored separately the effect of sex, and age on the pituitary gland volume in all our participants while controlling only for total ICV. We also separately examined the interaction between our groups and sex.

## Results

### Demographic and clinical data

Table 1 reports that the groups significantly differed on sex, race, site distribution, CPZ equivalents, clinical symptom severity, cognitive impairments level and duration of illness.

### Between-group comparisons

**Figure 1.**
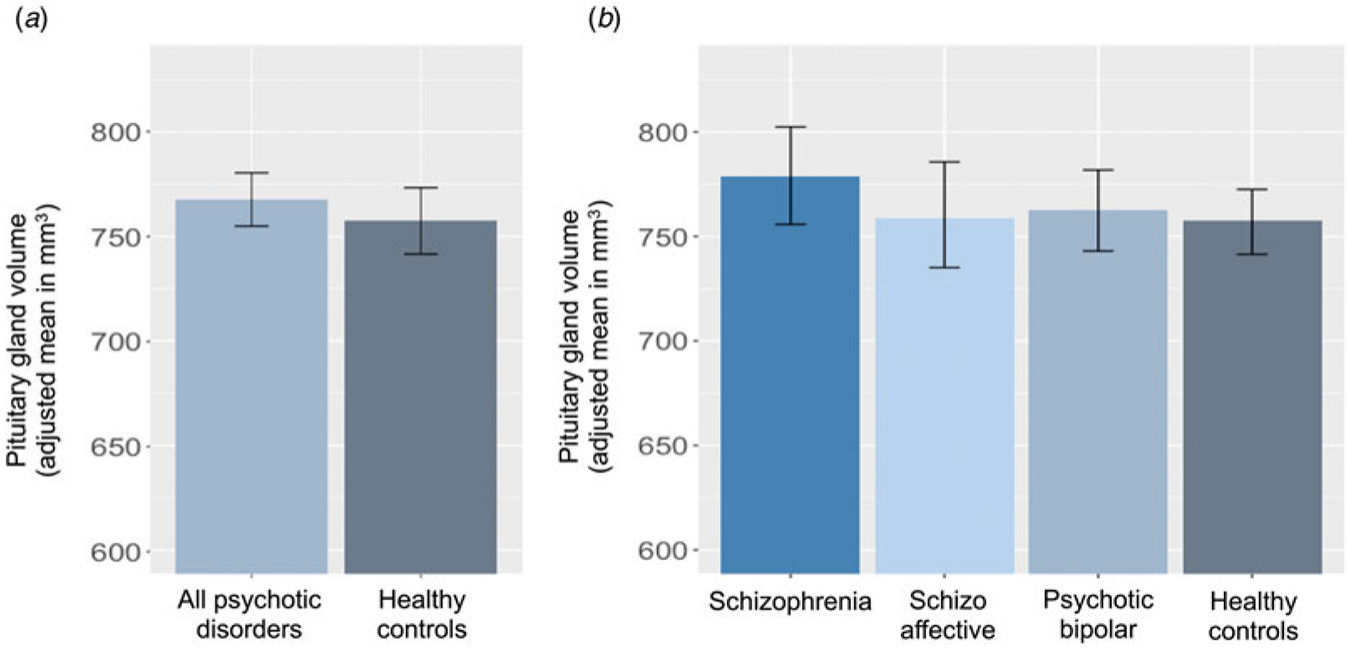
Pituitary gland volume between-group comparison (a) All patients with psychosis and healthy controls. (b) Each clinical diagnosis and healthy controls.

Figure 1 depicts that there was no significant difference in the mean pituitary gland volume between individuals diagnosed with psychotic disorders and healthy controls (*F*_(1, 735.88)_ = 1.04, *η*^*2*^ = 0.001, *p* = 0.31). When separated by clinical diagnosis, no statistical difference was observed between any groups (*F*_(3, 734.29)_ = 1.04, *η*^*2*^ = 0.004, *p* = 0.37). Table 2 presents all pairwise comparisons results.

### Factors influencing the pituitary gland volume in individuals with psychosis

**Figure 2.**
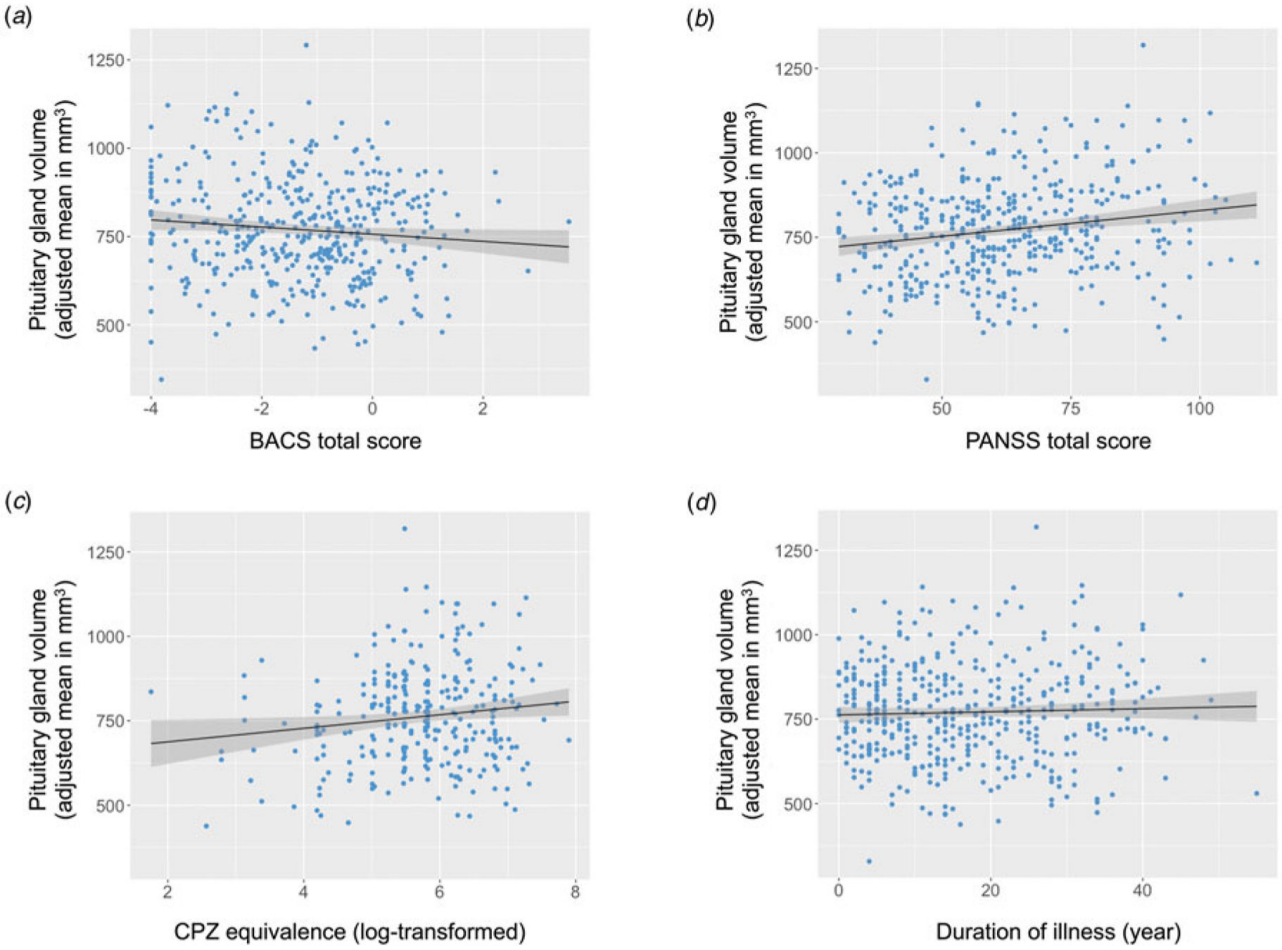
Associations between factors related to psychosis and pituitary gland volumes in individuals with psychotic disorders. (a) Cognition. (b) Symptom severity. (c) Antipsychotic medication dosage. (d) Duration of illness. Means were adjusted for age, sex, race, total intracranial volume, site, and scanner. BACS, Brief Assessment of Cognition in Schizophrenia Total Composite *Z* score; PANSS, Positive and Negative Syndrome Scale Total; CPZ, chlorpromazine equivalence.

The series of linear mixed effects models examining each predictor separately including age, sex, race, and total ICV as covariates, showed that lower cognition (*F*_(1, 423.54)_ = 4.76, *η^2^* = 0.01, *p* = 0.03), greater CPZ equivalents (*F*_(1, 274.02)_ = 5.20, *η*^*2*^ = 0.02, *p* = 0.02), and greater symptom severity (*F*_(1, 427.92)_ = 13.61, *η*^*2*^ = 0.03, *p* = 0.0002) were all significantly related to larger pituitary gland volumes in individuals with a diagnosis of psychotic disorder. Duration of illness was not significantly associated with pituitary gland volumes (*F*_(1, 425.43)_ = 0.82, *η*^*2*^ = 0.002, *p* = 0.36). Figure 2 illustrates the strength and direction of these associations.

When all variables were included in the same model (cognitive score, symptom severity, duration of illness and CPZ equivalents), including age, sex, race, and total ICV as covariates, only symptom severity showed a significant association with pituitary gland volumes (*F*_(1, 260.17)_ = 7.54, *η*^*2*^ = 0.03, *p* = 0.006).

We observed significant mild correlations between lower level of cognition (BACS score) and both higher symptom severity (*r* = 0.24, *p* < 0.0001) as well as higher CPZ equivalents (*r* = 0.16, *p* = 0.006). There was also a trend towards a significant correlation between symptoms severity and CPZ equivalents (*r* = 0.11, *p* = 0.07) (see online Supplementary Fig. S2). Post-hoc analysis shows no interaction between symptom severity effect on pituitary gland volumes and the different clinical diagnoses (*F*_(2, 420.82)_ = 0.50, *η*^*2*^ = 0.002, *p* = 0.60). Post-hoc analyses also confirm that all types of symptom severity (i.e. PANSS positive, PANSS negative, and PANSS general) were significantly related to larger pituitary gland volume (*all p*_*s*_ < 0.02). The relationship between CPZ equivalents and pituitary gland volumes was not statistically different whether patients were taking first or second generation antipsychotics (*F*_(1, 271.10)_ = 0.82, *η*^*2*^ = 0.003, *p* = 0.36). Furthermore, no significant differences on the pituitary gland volumes were observed between patients taking first *v.* second generation antipsychotics (*F*_(1, 273.18)_ = 1.96, *η*^*2*^ = 0.007, *p* = 0.16).

Exploratory analyses on the whole sample revealed no significant associations between age and the pituitary gland volumes (*F*_(1, 747)_ = 0.80, *η*^*2*^ = 0.001, *p* = 0.33), and a small but significant sex differences in pituitary gland volumes, with females showing greater volume compared to males (*F*_(1, 747)_ = 6.04, *η*^*2*^ = 0.008, *p* = 0.01), after correcting for total ICV. However, no significant interaction between our groups (patients and controls) and sex was observed in relation to pituitary gland volume (*F*_(1, 745)_ = 0.10, *η*^*2*^ = 0.0001, *p* = 0.75).

## Discussion

Psychotic disorders are among the leading causes of adverse life trajectories worldwide, and biomarkers could aid in their early detection (Kraguljac et al., 2021). This study is the first to investigate volume differences of the pituitary gland in a large transdiagnostic sample of individuals with psychotic disorders using an automatic segmentation pipeline. This well-powered sample allowed us to investigate a range of potential factors that could impact pituitary gland volume in this population. Enlargement of the pituitary gland volume was associated with various factors related to psychosis. Our main finding shows that when all factors are considered, only symptom severity significantly predicts pituitary gland volume. This suggests that pituitary gland function is more affected in individuals with more severe symptoms. This is in line with previous studies that have found an increase in pituitary gland volume to be linked to less improvement in psychotic symptoms in early psychosis (Garner et al., 2005; Takahashi et al., 2011). Interestingly, Premkumar et al. (2018) also reported that individuals with treatment-resistant chronic schizophrenia who received cognitive behavioral therapy (CBT) showed both a reduction in their pituitary volume and improvement in their clinical symptoms compared to those who did not receive the therapy. One hypothesis advanced by the authors is that CBT could reduce the impact of stress, therefore lowering cortisol levels and improving HPA axis regulation, which may ultimately reduce pituitary volume in this population.

Contrary to our hypothesis, pituitary gland volumes were not significantly different between individuals with psychosis and healthy controls, or between any of the diagnostic groups. These findings support previous studies reporting no significant differences in pituitary gland volumes between individuals with psychosis and healthy controls (Habets et al., 2012; Klomp et al., 2012; Nicolo et al., 2010). They also corroborate the meta-analysis by Nordholm et al. (2013) in which the only finding was a trend-level difference in high-risk individuals who developed psychosis and in individuals experiencing a first episode of psychosis when compared to controls, and not in individuals with enduring schizophrenia. Taken together, these results suggest that an increased pituitary volume may reflect a state-related vulnerability in the trajectory of psychotic symptoms development, and in individuals who are more severely symptomatic. However, this does not seem to be linked to duration of illness. No significant relationship between the pituitary gland and the duration of illness was observed, which is in line with the results reported by Klomp et al. (2012).

Cognitive performance was negatively associated with pituitary volume enlargement in our group of individuals living with psychotic disorders. Studies have previously demonstrated that abnormal HPA axis functioning is associated with distorted cortisol responses which is also linked to cognitive impairments in individuals with schizophrenia (Aas et al., 2011; Walder et al., 2000). Evidence also shows that pituitary volume enlargement in individuals with schizophrenia is negatively associated with cognitive performance as demonstrated through the BACS memory test (Takahashi et al., 2018). Therefore, pituitary volume enlargement could reflect HPA axis hyperactivity associated with cognitive deficits in individuals living with psychosis. In the current study, cognitive performances also showed a mild but significant correlation with symptoms severity in patients. This is particularly interesting given that individuals with global cognitive difficulty are also at greater risk of developing psychosis (Cornblatt, Obuchowski, Roberts, Pollack, & Erlenmeyer-Kimling, 1999; Snitz, MacDonald, & Carter, 2006). Further research is needed to better understand the relationship between pituitary gland volume and cognitive performance in both individuals with psychosis and those that are at risk of developing psychosis.

Our findings also demonstrate a significant positive association between greater antipsychotic dose (as measured by CPZ equivalents) and pituitary volume enlargement in our sample of individuals living with psychotic disorders. This relationship is present in both groups of patients who used typical and atypical antipsychotics. These findings are in line with previous evidence demonstrating the enlarging effect of antipsychotic medication on total pituitary volume (Klomp et al., 2012; MacMaster et al., 2007). Takahashi et al. (2018) also show that individuals with schizophrenia treated with antipsychotic medication have significantly larger pituitary volume compared to antipsychotic-free patients which includes individuals with first episode and chronic schizophrenia. However, antipsychotic-free patients also have a larger pituitary volume in comparison to healthy controls, which suggests that antipsychotic medication alone may not solely lead to the enlargement of pituitary volume (Takahashi et al., 2018). Additionally, it is plausible that individuals with more pronounced symptoms may receive higher doses of antipsychotic medication, and thus the association between CPZ equivalents and pituitary gland volume may be coincidental. In our sample, only a small trend towards a significant correlation was observed between CPZ equivalents and symptoms severity, however it is possible that antipsychotic doses may serve as a proxy of how severely ill our participants are.

Our findings suggest that pituitary volume enlargement can serve as a distinct characteristic of vulnerability to psychosis, which can be influenced by several factors, including clinical symptom severity (assessed using the PANSS total score), and cognitive symptoms severity (assessed through the BACS), as well as CPZ equivalents (Cohrs et al., 2006; Takahashi et al., 2018; Takahashi & Suzuki, 2018; Walker, Mittal, & Tessner, 2008). This provides a possible explanation for the inconsistent findings observed in previous studies which may have varied on these characteristics. In addition, studies have reported differences in pituitary volume across different stages of the illness which could also have contributed to the large variability among previous findings (Borges et al., 2013; Pariante et al., 2004).

Studies have reported that individuals with first episode schizophrenia had a larger pituitary gland volume compared to individuals with established schizophrenia which could be associated with hyperactivity of the HPA axis in earlier and more symptomatic phases (Pariante et al., 2004; Takahashi et al., 2018; Upadhyaya et al., 2007). Pituitary gland volume enlargement has also been observed among high-risk individuals who later transitioned to developing psychosis (Saunders et al., 2019; Shah et al., 2015). This suggests that pituitary gland volume enlargement could precede the onset of psychosis and may become more pronounced among those who develop more severe psychotic symptoms. The lack of significant differences between our patient cohort and controls provide further support for this notion as most of our cohort consisted of relatively stable patients that could have regained equilibrium in their pituitary gland volume. This contrasts with other studies that have investigated patients that were more acutely unwell when scanned (i.e., at-risk mental state or first episode psychosis) and observed mixed findings (Borges et al., 2013; Büschlen et al., 2011; Garner et al., 2005). Enlarged pituitary gland could be a state marker for symptom severity, not only in individuals with psychotic disorders, but also in high-risk populations. Hence, studies using larger samples in high-risk populations are warranted.

Interestingly, altered release of hormones that are regulated by the pituitary gland, such as cortisol and prolactin has been implicated in psychotic disorders (Borges et al., 2013). Individuals with psychotic disorders experience HPA axis hyperactivity, which may be linked to stress vulnerability and higher levels of cortisol (Mitropoulou et al., 2004; Walker et al., 2008). Hormonal dysregulation in the course of the illness could also lead to changes in pituitary gland volume (Aiello et al., 2012; Walker et al., 2008). It is therefore possible that more symptomatic individuals with psychotic disorders have a higher vulnerability to stress, but further studies investigating stress, cortisol, and the pituitary gland are warranted. Furthermore, studies have shown that increased pituitary volume is associated with increased emotional stress reactivity in individuals with psychotic disorders, their sibling, and healthy controls, suggesting that pituitary volume may be considered as a non-specific measure of stress (Habets et al., 2012). Future studies should determine whether the association between pituitary volume and symptom severity is specific to psychosis or if it might also indicate general stress in non-psychotic conditions. Future longitudinal research is also needed to better understand the relationship between hormonal dysregulation, pituitary gland volume, and psychotic disorders.

Another factor that could contribute to the enlargement of the pituitary gland volume and indicate HPA axis hyperactivity in individuals with psychosis is the possibility of higher antipsychotic medication dosages (Pariante et al., 2004). Longitudinal studies examining the influence of antipsychotics on HPA axis activity and pituitary gland volumes in antipsychotic-naive patients are warranted. Furthermore, future studies should explore whether the enlargement of the pituitary gland is influenced by factors such as HPA axis hyperactivity or hypercortisolemia, distinct from medication-related hyperprolactinemia. Investigating these aspects could offer valuable insights into the underlying mechanisms of enlarged pituitary glands.

### Limitations

The current study did not investigate the impact of pituitary hormones on psychosis, which limits its conclusions. Future large-scale studies should examine the effect of pituitary hormones on the development of psychosis. Additionally, further research is needed to gain a deeper understanding of the relationship between stress impact in individuals with psychosis, symptom severity, and the pituitary gland. In the future, these findings may assist in the development of more personalized and targeted treatments for these individuals. Furthermore, the specific relationship between age and duration of illness warrants further investigation with subgroup analyses (i.e. non-linear models) as that could lead to more insightful findings, especially in studies with large cohorts. Lastly, we are missing information in about a third of our sample regarding their medication intake.

Raw data on CPZ equivalents were also skewed and required log-transformation to conduct the analyses performed in the study. Therefore, the results regarding the impact of CPZ equivalents on the pituitary gland should be interpreted with caution and aim to be replicated. Future studies should also more thoroughly characterize information about antipsychotic medication and explore the potential impact of prolactin sparing/raising medication on the pituitary gland in this population. We also had to exclude many participants from the analyses due to segmentation failure. It is notably challenging to segment the pituitary gland compared to other structures like the hippocampus, because of its small size (Mlynarski, Delingette, Alghamdi, Bondiau, & Ayache, 2020). Hence, it was essential to meticulously review each segmentation and ensure that only successful ones were included in our study.

Importantly, our fully automated segmentation pipeline demonstrated high performance, accurately delineating the intended structures in approximately 90% of our samples. The current fully automated segmentation pipeline is different from previous pituitary gland segmentation methods used in individuals with psychosis that used manual and semi-automatic segmentation methods. It is therefore possible that some methods may be more or less sensitive, and this can also explain some of the inconsistencies in findings across studies. Furthermore, lifetime cumulative doses of antipsychotic medication were not collected. This should be considered in future research considering its potential greater impact on the brain anatomy and function among this population (Husa et al., 2017). Lastly, due to the transdiagnostic nature of our sample, the 30-item PANSS was administered to all our participants to measure psychotic symptoms severity. As this scale has been mainly validated in individuals with schizophrenia and schizoaffective disorder, it is possible that this may limit the generalizability of our results to our participants with bipolar disorders (Anderson et al., 2017).

## Conclusions

The current study is the first to investigate pituitary volume in a large sample of individuals with transdiagnostic psychotic disorders. Our findings provide a unique insight on the factors that can influence pituitary gland volume in this population. When compared to controls, individuals with psychotic disorders do not show significant differences in pituitary gland volume. Symptom severity, cognitive functioning, and CPZ equivalents, all seem to have an impact on the pituitary gland volume among individuals living with psychotic disorders. When all these variables were considered, however, only symptom severity has a significant impact. Our findings help clarify previous inconsistent reports and support further investigation of stress vulnerability biomarkers in psychosis and high-risk populations. These findings suggest that a larger pituitary gland volume is not a trait biomarker for psychosis, but may serve as a state-related biomarker linked with severe symptoms. To confirm this, further longitudinal studies in individuals with psychosis are necessary to determine if increased pituitary gland volume correlates with increased symptom severity over time.

## Supplementary Material

supplementary material

## Figures and Tables

**Table 1.** Demographic and clinical data

	Schizophrenia	Schizoaffective	Psychotic bipolar	Healthy controls	*p*-value
Total (*n*)	174	114	167	296	
Age (mean [s.d.])	34.21 (12.48)	34.84 (11.38)	35.47 (12.83)	35.98 (12.25)	0.483
Sex F (*n* [%])	54 (31.0)	62 (54.4)	113 (67.7)	168 (56.9)	<0.001
Race (*n* [%])					<0.001
AA	75 (43.4)	43 (37.7)	29 (17.4)	80 (27.1)	
AE	1 (0.6)	0 (0.0)	0 (0.0)	1 (0.3)	
AS	2 (1.2)	1 (0.9)	4 (2.4)	14 (4.7)	
CA	86 (49.7)	65 (57.0)	129 (77.2)	187 (63.4)	
MR	3 (1.7)	5 (4.4)	1 (0.6)	6 (2.0)	
NH	0 (0.0)	0 (0.0)	0 (0.0)	1 (0.3)	
OT	6 (3.5)	0 (0.0)	4 (2.4)	6 (2.0)	
Site (n [%])					<0.001
Dallas	12 (6.9)	27 (23.7)	18 (10.8)	47 (15.9)	
Hartford	26 (14.9)	31 (27.2)	30 (18.0)	57 (19.3)	
Baltimore	62 (35.6)	25 (21.9)	27 (16.2)	51 (17.2)	
Chicago	30 (17.2)	20 (17.5)	48 (28.7)	61 (20.6)	
Detroit	10 (5.7)	5 (4.4)	13 (7.8)	40 (13.5)	
Boston	34 (19.5)	6 (5.3)	31 (18.6)	40 (13.5)	
CPZ (mean [s.d.])	487.55 (381.37)	515.86 (471.64)	349.60 (328.96)	N/A	0.010
Duration of illness (mean [s.d.])	14.99 (11.64)	18.29 (11.22)	18.39 (11.96)	N/A	0.014
PANSS (mean [s.d.])	66.25 (17.39)	69.33 (16.01)	52.75 (13.74)	N/A	<0.001
BACS (mean [s.d.])	−1.74 (1.40)	−1.43 (1.46)	−0.81 (1.32)	0.02 (1.20)	<0.001

s.d., Standard deviation; F, female; CA, Caucasian; AA, African American; AE, American Indian; AS, Asian; MR, Mixed Race; NH, Native Hawaiian; OT, Other; BACS, Brief Assessment of Cognition in Schizophrenia Total Composite Z score; PANSS, Positive and Negative Syndrome Scale Total; CPZ, chlorpromazine equivalents.

*Note.* Information is missing for the following variables: sex (*n* = 1 control); race (*n* = 1 control); duration of illness (*n* = 17 patients); PANSS (*n* = 17 patients); CPZ (*n* = 168 patients); BACS (*n* = 18 controls and *n* = 19 patients).

**Table 2.** Post-hoc pairwise comparisons

Contrast	Estimate	s.e.	*df*	*T*	*p* value
C – PBD	0.03738	0.0907	733	0.412	0.9764
C – SADP	−0.00608	0.1037	734	−0.059	0.9999
C – SZP	−0.15715	0.0930	735	−1.689	0.3300
SADP – SZP	−0.15106	0.1152	735	−1.311	0.5561
PBD – SADP	0.03130	0.1158	734	0.270	0.9931
PBD– SZP	−0.11976	0.1064	733	−1.125	0.6741

*Note.* C, controls; PBD, psychotic bipolar disorder; SADP, schizoaffective disorder; SZP, schizophrenia disorder; s.e., standard error; df, degree of freedom.

All *p* values are corrected for multiple comparisons (family-wise error).

## References

[R1] Aas M, Dazzan P, Mondelli V, Toulopoulou T, Reichenberg A, Di Forti M, … Pariante CM (2011). Abnormal cortisol awakening response predicts worse cognitive function in patients with first-episode psychosis. Psychological Medicine, 41(3), 463–476. 10.1017/S0033291710001170 20529412 PMC3513413

[R2] Aiello G, Horowitz M, Hepgul N, Pariante CM, & Mondelli V (2012). Stress abnormalities in individuals at risk for psychosis: A review of studies in subjects with familial risk or with “ at risk” mental state. Psychoneuroendocrinology, 37(10), 1600–1613. 10.1016/j-psyneuen.2012.05.003 22663896

[R3] Anderson AE, Mansolf M, Reise SP, Savitz A, Salvadore G, Li Q, & Bilder RM (2017). Measuring pathology using the PANSS across diagnoses: Inconsistency of the positive symptom domain across schizophrenia, schizoaffective, and bipolar disorder. Psychiatry Research, 258, 207–216. 10.1016/j.psychres.2017.08.009 28899614 PMC5681392

[R4] Borges S, Gayer-Anderson C, & Mondelli V (2013). A systematic review of the activity of the hypothalamic–pituitary–adrenal axis in first episode psychosis. Psychoneuroendocrinology, 38(5), 603–611. 10.1016/j.psyneuen.2012.12.025 23369532

[R5] Büschlen J, Berger GE, Borgwardt SJ, Aston J, Gschwandtner U, Pflueger MO, … Riecher-Rössler A (2011). Pituitary volume increase during emerging psychosis. Schizophrenia Research, 125(1), 41–48. 10.1016/j.schres.2010.09.022 21074369

[R6] Chakravarty MM, Steadman P, van Eede MC, Calcott RD, Gu V, Shaw P, … Lerch JP (2013). Performing label-fusion-based segmentation using multiple automatically generated templates. Human Brain Mapping, 34(10), 2635–2654. 10.1002/hbm.22092 22611030 PMC4896505

[R7] Clark IA, Mackay CE, & Goodwin GM (2014). Pituitary gland volumes in bipolar disorder. Journal of Affective Disorders, 169, 197–202. 10.1016/j.jad.2014.08.022 25212995

[R8] Cohrs S, Röher C, Jordan W, Meier A, Huether G, Wuttke W, … Rodenbeck A (2006). The atypical antipsychotics olanzapine and quetiapine, but not haloperidol, reduce ACTH and cortisol secretion in healthy subjects. Psychopharmacology, 185(1), 11–18. 10.1007/s00213-005-0279-x 16432682

[R9] Cornblatt B, Obuchowski M, Roberts S, Pollack S, & Erlenmeyer-Kimling L (1999). Cognitive and behavioral precursors of schizophrenia. Development and Psychopathology, 11(3), 487–508. 10.1017/S0954579499002175 10532621

[R10] Cullen AE, Zunszain PA, Dickson H, Roberts RE, Fisher HL, Pariante CM, & Laurens KR (2014). Cortisol awakening response and diurnal cortisol among children at elevated risk for schizophrenia: Relationship to psychosocial stress and cognition. Psychoneuroendocrinology, 46, 1–13. 10.1016/j.psyneuen.2014.03.010 24882153 PMC4065330

[R11] Delvecchio G, Altamura AC, Soares JC, & Brambilla P (2017). Pituitary gland in bipolar disorder and major depression: Evidence from structural MRI studies: Special section on “translational and neuroscience studies in affective disorders”, section editor, Maria Nobile MD, PhD. This section of JAD focuses on the re. Journal of Affective Disorders, 218, 446–450. 10.1016/j.jad.2017.03.066 28412090

[R12] Delvecchio G, Mandolini GM, Perlini C, Barillari M, Marinelli V, Ruggeri M, … Brambilla P (2018). Pituitary gland shrinkage in bipolar disorder: The role of gender. Comprehensive Psychiatry, 82, 95–99. 10.1016/j.comppsych.2018.01.014 29454165

[R13] Egger J, Kapur T, Fedorov A, Pieper S, Miller JV, Veeraraghavan H, … Kikinis R (2013). GBM Volumetry using the 3D sheer medical image computing platform. Scientific Reports, 3, 1364. 10.1038/srep01364 23455483 PMC3586703

[R14] Garner B, Pariante CM, Wood SJ, Velakoulis D, Phillips L, Soulsby B, … Pantelis C (2005). Pituitary volume predicts future transition to psychosis in individuals at ultra-high risk of developing psychosis. Biological Psychiatry, 58, 417–423. 10.1016/j-biopsych.2005.04.018 16026767

[R15] Guilera G, Gómez-Benito J, Pino O, Rojo JE, Cuesta MJ, Martínez-Arán A, … Rejas J (2012). Utility of the World Health Organization Disability Assessment Schedule II in schizophrenia. Schizophrenia Research, 138(2–3), 240–247. 10.1016/j.schres.2012.03.031 22521724

[R16] Gurok MG, Keles DD, Korkmaz S, Yildirim H, Kilic Mç, & Atmaca M (2019). Smaller pituitary volumes in patients with delusional disorder. Medical Archives (Sarajevo, Bosnia and Herzegovina), 73(4), 253–256. 10.5455/medarh.2019.73.253-256 31762560 PMC6853737

[R17] Habets P, Collip D, Myin-Germeys I, Gronenschild E, Van Bronswijk S, Hofman P, … Marcelis M (2012). Pituitary volume, stress reactivity and genetic risk for psychotic disorder. Psychological Medicine, 42(7), 1523–1533. 10.1017/S0033291711002728 22130309

[R18] Hill SK, Reilly JL, Keefe RSE, Gold JM, Bishop JR, Gershon ES, … Sweeney JA (2013). Neuropsychological impairments in schizophrenia and psychotic bipolar disorder: Findings from the Bipolar-Schizophrenia Network on Intermediate Phenotypes (B-SNIP) study. American Journal of Psychiatry, 170(11), 1275–1284. 10.1176/appi.ajp.2013.12101298 23771174 PMC5314430

[R19] Husa AP, Moilanen J, Murray GK, Marttila R, Haapea M, Rannikko I, … Jääskeläinen E (2017). Lifetime antipsychotic medication and cognitive performance in schizophrenia at age 43 years in a general population birth cohort. Psychiatry Research, 247, 130–138. 10.1016/j.psychres.2016.10.085 27888683 PMC5241225

[R20] Kay SR, Fiszbein A, & Opler LA (1987). The positive and negative syndrome scale (PANSS) for schizophrenia. Schizophrenia Bulletin, 13(2), 261–276. 10.1093/schbul/13.2.261 3616518

[R21] Keefe RS, Goldberg TE, Harvey PD, Gold JM, Poe MP, & Coughenour L (2004). The Brief Assessment of Cognition in Schizophrenia: Reliability, sensitivity, and comparison with a standard neurocognitive battery. Schizophrenia Research, 68(2–3), 283–297. 10.1016/j.schres.2003.09.011 15099610

[R22] Kelly S, Guimond S, Lyall A, Stone WS, Shenton ME, Keshavan M, & Seidman LJ (2019). Neural correlates of cognitive deficits across developmental phases of schizophrenia. Neurobiology of Disease, 131(10435), 3. 10.1016/j.nbd.2018.12.013 30582983

[R23] Klomp A, Koolschijn PCMP, HulshoffPol HE, Kahn RS, & Van Haren NEM (2012). Hypothalamus and pituitary volume in schizophrenia: A structural MRI study. International Journal of Neuropsychopharmacology, 15 (2), 281–288. 10.1017/S1461145711000794 21733239

[R24] Kraguljac NV, McDonald WM, Widge AS, Rodriguez CI, Tohen M, & Nemeroff CB (2021). Neuroimaging biomarkers in schizophrenia. The American Journal of Psychiatry, 178(6), 509–521. 10.1176/appi.ajp.2020.20030340 33397140 PMC8222104

[R25] Kuznetsova A, Brockhoff PB, & Christensen RHB (2017). lmerTest package: Tests in linear mixed effects models. Journal of Statistical Software, 82(13), 1–26. 10.18637/JSS.V082.I13

[R26] MacMaster FP, El-Sheikh R, Upadhyaya AR, Nutche J, Rosenberg DR, & Keshavan M (2007). Effect of antipsychotics on pituitary gland volume in treatment-naïve first-episode schizophrenia: A pilot study. Schizophrenia Research, 92(1–3), 207–210. 10.1016/j.schres.2007.01.022 17337162

[R27] Mitropoulou V, Goodman M, Sevy S, Elman I, New AS, Iskander EG, … Siever LJ (2004). Effects of acute metabolic stress on the dopaminergic and pituitary–adrenal axis activity in patients with schizotypal personality disorder. Schizophrenia Research, 70(1), 27–31. 10.1016/j.schres.2003.10.008 15246460

[R28] Mlynarski P, Delingette H, Alghamdi H, Bondiau P-Y, & Ayache N (2020). Anatomically consistent CNN-based segmentation of organs-at-risk in cranial radiotherapy. Journal of Medical Imaging, 7(01), 1. 10.1117/1.jmi.7.1.014502 PMC7016364 32064300

[R29] Nicolo JP, Berger GE, Garner BA, Velakoulis D, Markulev C, Kerr M, … Wood SJ (2010). The effect of atypical antipsychotics on pituitary gland volume in patients with first-episode psychosis: A longitudinal MRI study. Schizophrenia Research, 116(1), 49–54. 10.1016/j.schres.2009.10.005 19896337

[R30] Nordholm D, Krogh J, Mondelli V, Dazzan P, Pariante C, & Nordentoft M (2013). Pituitary gland volume in patients with schizophrenia, subjects at ultra high-risk of developing psychosis and healthy controls: A systematic review and meta-analysis. Psychoneuroendocrinology, 38(11), 2394–2404. 10.1016/j.psyneuen.2013.06.030 23890984

[R31] Pariante CM, Dazzan P, Danese A, Morgan KD, Brudaglio F, Morgan C, … Murray RM (2005). Increased pituitary volume in antipsychotic-free and antipsychotic-treated patients of the Æsop first-onset psychosis study. Neuropsychopharmacology, 30(10), 1923–1931. 10.1038/sj.npp.1300766 15956995

[R32] Pariante CM, Vassilopoulou K, Velakoulis D, Phillips L, Soulsby B, Wood SJ, … Pantelis C (2004). Pituitary volume in psychosis. British Journal of Psychiatry, 185, 5–10. 10.1192/bjp.185.1.5 15231549

[R33] Park MTM, Pipitone J, Baer LH, Winterburn JL, Shah Y, Chavez S, … Chakravarty MM (2014). Derivation of high-resolution MRI atlases of the human cerebellum at 3 T and segmentation using multiple automatically generated templates. NeuroImage, 95, 217–231. 10.1016/j.neuroimage.2014.03.037 24657354

[R34] Peper JS, Brouwer RM, van Leeuwen M, Schnack HG, Boomsma DI, Kahn RS, & Hulshoff Pol HE (2010). HPG-axis hormones during puberty: A study on the association with hypothalamic and pituitary volumes. Psychoneuroendocrinology, 35(1), 133–140. 10.1016/j.psyneuen.2009.05.025 19570613

[R35] Premkumar P, Bream D, Sapara A, Fannon D, Anilkumar AP, Kuipers E, & Kumari V (2018). Pituitary volume reduction in schizophrenia following cognitive behavioural therapy. Schizophrenia Research, 192, 416–422. 10.1016/j.schres.2017.04.035 28434719 PMC5821679

[R36] Sassi RB, Nicoletti M, Brambilla P, Harenski K, Mallinger AG, Frank E, … Soares JC (2001). Decreased pituitary volume in patients with bipolar disorder. Biological Psychiatry, 50(4), 271–280. 10.1016/S0006-3223(01)01086-1 11522262

[R37] Saunders TS, Mondelli V, & Cullen AE (2019). Pituitary volume in individuals at elevated risk for psychosis: A systematic review and meta-analysis. Schizophrenia Research, 213, 23–31. 10.1016/j.schres.2018.12.026 30600112

[R38] Shah JL, Tandon N, Howard ER, Mermon D, Miewald JM, Montrose DM, & Keshavan MS (2015). Pituitary volume and clinical trajectory in young relatives at risk for schizophrenia. Psychological Medicine, 45(13), 2813–2824. 10.1017/S003329171500077X 26149540

[R39] Snitz BE, MacDonald AW, & Carter CS (2006). Cognitive deficits in unaffected first-degree relatives of schizophrenia patients: A meta-analytic review of putative endophenotypes. Schizophrenia Bulletin, 32(1), 179–194. 10.1093/schbul/sbi048 16166612 PMC2632195

[R40] Soni BK, Joish UK, Sahni H, George RA, Sivasankar R, & Aggarwal R (2017). A comparative study of pituitary volume variations in MRI in acute onset of psychiatric conditions. Journal of Clinical and Diagnostic Research, 11(2), TC01–TC04. 10.7860/JCDR/2017/23585.9330 PMC5376881 28384955

[R41] Takahashi T, Higuchi Y, Komori Y, Nishiyama S, Takayanagi Y, Sasabayashi D, … Suzuki M (2018). Pituitary volume and socio-cognitive functions in individuals at risk of psychosis and patients with schizophrenia. Frontiers in Psychiatry, 9, 574. 10.3389/fjpsyt.2018.00574 30473669 PMC6237858

[R42] Takahashi T, Nakamura K, Nishiyama S, Furuichi A, Ikeda E, Kido M, … Suzuki M (2013). Increased pituitary volume in subjects at risk for psychosis and patients with first-episode schizophrenia. Psychiatry and Clinical Neurosciences, 67(7), 540–548. 10.1111/pcn.12093 24102999

[R43] Takahashi T, & Suzuki M (2018). Brain morphologic changes in early stages of psychosis: Implications for clinical application and early intervention. Psychiatry and Clinical Neurosciences, 72(8), 556–571. 10.1111/pcn.12670 29717522

[R44] Takahashi T, Suzuki M, Velakoulis D, Lorenzetti V, Soulsby B, Zhou SY, … Pantelis C (2009). Increased pituitary volume in schizophrenia spectrum disorders. Schizophrenia Research, 108(1–3), 114–121. 10.1016/j.schres.2008.12.016 19162445

[R45] Takahashi T, Zhou SY, Nakamura K, Tanino R, Furuichi A, Kido M, … Suzuki M (2011). Longitudinal volume changes of the pituitary gland in patients with schizotypal disorder and first-episode schizophrenia. Progress in Neuro-Psychopharmacology and Biological Psychiatry, 35(1), 177–183. 10.1016/j.pnpbp.2010.10.023 21044655

[R46] Tamminga CA, Pearlson G, Keshavan M, Sweeney J, Clementz B, & Thaker G (2014). Bipolar and schizophrenia network for intermediate phenotypes: Outcomes across the psychosis continuum. Schizophrenia Bulletin, 40(SUPPL. 2), 131–137. 10.1093/schbul/sbt179 PMC3934403 24562492

[R47] Upadhyaya AR, El-Sheikh R, MacMaster FP, Diwadkar VA, & Keshavan MS (2007). Pituitary volume in neuroleptic-naive schizophrenia: A structural MRI study. Schizophrenia Research, 90(1–3), 266–273. 10.1016/j.schres.2006.09.033 17187962

[R48] Walder DJ, Walker EF, & Lewine RJ (2000). Cognitive functioning, cortisol release, and symptom severity in patients with schizophrenia. Biological Psychiatry, 48(12), 1121–1132. 10.1016/S0006-3223(00)01052-0 11137052

[R49] Walker E, Mittal V, & Tessner K (2008). Stress and the hypothalamic pituitary adrenal axis in the developmental course of schizophrenia. Annual Review of Clinical Psychology, 4, 189–216. 10.1146/annurev.clinpsy.4.022007.141248 18370616

[R50] Wong APY, Pipitone J, Park MTM, Dickie EW, Leonard G, Perron M, … Paus T (2014). Estimating volumes of the pituitary gland from T1-weighted magnetic-resonance images: Effects of age, puberty, testosterone, and estradiol. NeuroImage, 94, 216–221. 10.1016/j.neuroimage.2014.02.030 24632090

