# Supplementary material for: Enlarged pituitary gland volume: a possible state rather than trait marker of psychotic disorders
Source: Psychol Med. Author manuscript; Available in PMC 2025 Jun 1. (PMC11132920; doi:10.1017/S003329172300380X)
Supplement: supplementary material [file NIHMS1983205-supplement-supplementary_material.docx]

**Supplementary Material**

*Site-Specific Imaging Acquisition Parameters*

All sites followed the Alzheimer's Disease Neuroimaging Initiative (ADNI1) protocol (see http://adni.loni.usc.edu/methods/documents/mri-protocols/ for full list of parameters). The MPRAGE or IR-SPGR parameters at each site, as appropriate for each scanner brand or model, were as follows:

Baltimore, Siemens Trio (Maryland Psychiatric Research Center, University of Maryland, Baltimore MD): 3D acquisitions, sagittal slab, shot interval 2300ms, inversion time 900ms, TR 6.8ms, TE 2.91ms, flip angle 9º, FOV 256 (foot-to-head) x 240 (anterior-to-posterior) mm2, matrix 256x240, in-pane resolution 1x1 mm2, 160 slices, slice thickness 1.2 mm, voxel size 1x1x1.2mm3, total scan duration 9min 14sec.

Boston, GE Signa HDxt (Harvard Medical School, Boston MA): 3D acquisitions, sagittal slab, inversion time 650ms, TR 7.0ms, TE 3.0ms, flip angle 8º, FOV 256 (foot-to-head) x 256 (anterior-to-posterior) mm2, matrix 256x256, in-pane resolution 1x1 mm2, 166 slices, slice thickness 1.2 mm, voxel size 1x1x1.2mm3, total scan duration 9min 58sec.

Chicago, GE Signa (University of Chicago, Chicago IL): 3D acquisitions, sagittal slab, shot interval 2300ms, inversion time 700ms, TR 6.99ms, TE 2.85ms, flip angle 8º, FOV 260 (foot-to-head) x 260 (anterior-to-posterior) mm2, matrix 256x256, in-plane resolution 1x1 mm2, 166 slices, slice thickness 1.2 mm, voxel size 1x1x1.2mm3, total scan duration 10min 28sec.

Dallas, Philips Achieva (UT Southwestern Medical Center, Dallas TX): 3D acquisitions, sagittal slab, shot interval 3000ms, inversion time 846ms, TR 6.8ms, TE 3.1ms, flip angle 8º, FOV 256 (foot-to-head) x 240 (anterior-to-posterior) mm2, matrix 256x240, in-plane resolution 1x1 mm2, 170 slices, slice thickness 1.2 mm, voxel size 1x1x1.2mm3, total scan duration 9min 19sec.

Detroit, Siemens Trio (Wayne State University, Detroit MI): 3D acquisitions, sagittal slab, shot interval 2300ms, inversion time 900ms, TR 6.8ms, TE 2.74ms, flip angle 8º, FOV 176 (foot-to-head) x 256 (anterior-to-posterior) mm2, matrix 176x256x176, in-pane resolution 1x1 mm2, slice thickness 1.2mm, voxel size 1x1x1.2mm3, total scan duration 10min 09sec.

Hartford, Siemens Allegra (Olin Institute of Living, Hartford Hospital, Hartford CT): 3D acquisitions, sagittal slab, shot interval 2300ms, inversion time 900ms, TR 7.2ms, TE 2.91ms, flip angle 9º, FOV 256 (foot-to-head) x 240 (anterior-to-posterior) mm2, matrix 256x240, in-pane resolution 1x1 mm2, 160 slices, slice thickness 1.2mm, voxel size 1x1x1.2mm3, total scan duration 9min 14sec.

*Pituitary gland segmentation*


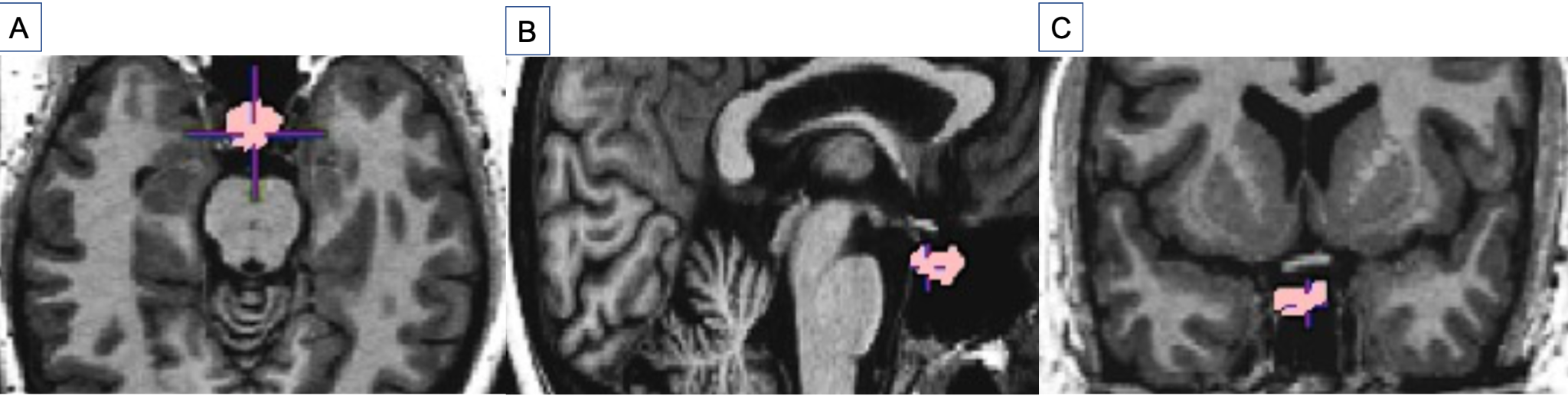
**Supplementary Fig 1.** Example of Pituitary Gland Segmentation Conducted on a Study Participant


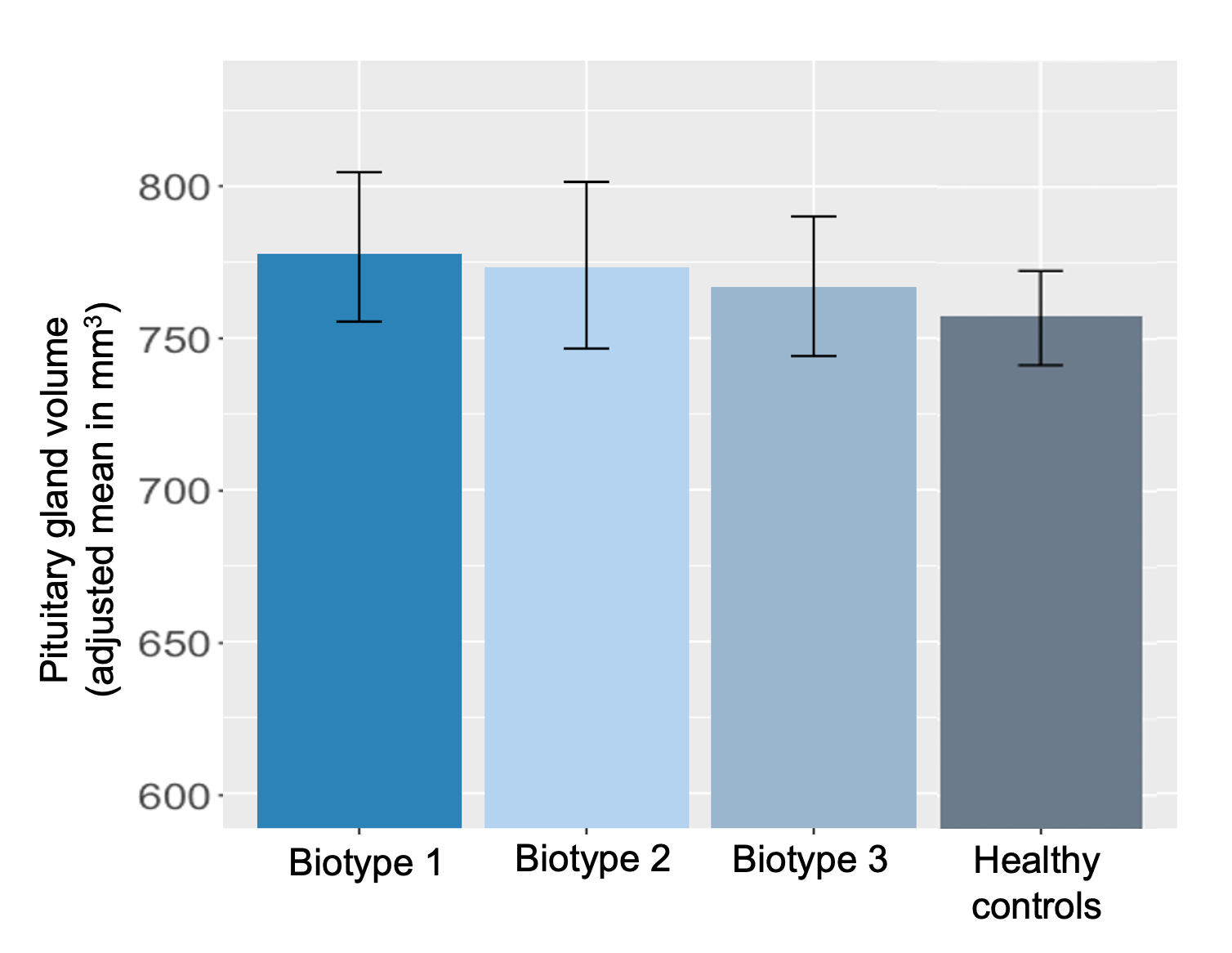
 **Supplementary Fig 2.** Adjusted Mean Pituitary Gland Volume Across Biotypes and Healthy Controls.

Post-hoc analysis shows no interaction between symptom severity effect on pituitary gland volumes and the different clinical diagnoses (*F*_(2, 420.82)_ = 0.50, *η²* = 0.002, *p* = .60). nor the Biotypes (*F*_(2, 366.95)_ = 0.84, *η²* = 0.004, *p* = .43).Visual inspection were performed on each automatized segmentation by YT examining sagittal, coronal, and axial view. When part of the pituitary gland was missed or the segmentation included regions outside of the pituitary gland, we excluded this participant from our analysis (n=97). No site was more represented in those that we had to exclude.

*Biotype classification*

Clementz and colleagues provide a comprehensive method for biotype classification, which involves various tasks completed by participants, such as BACS (Keefe et al., 2004), stop-signal, pro- and anti-saccade, and auditory paired stimulus and oddball tasks. After quality control of the data and excluding participants who did not receive a Biotype classification, a total of three hundred ninety-three participants with psychotic disorders were included for the Biotype between-group analysis in the study (138 Biotype-1, 122 Biotype-2, and 133 Biotype-3). The methods for each task were published using participants from the first iteration of the B-SNIP project (B-SNIP1) and subsequently replicated with the PARDIP and B-SNIP2 sample (Clementz et al., 2016; Ethridge et al., 2017, 2014; Gotra et al., 2020; Hamm et al., 2014; S Kristian Hill et al., 2013; Huang et al., 2022; Parker et al., 2020, 2021; Reilly et al., 2014). Within each task, a principal components analysis was conducted based on the full combined sample (healthy and psychosis). The resulting components were referred to as "Biofactors" since they captured multiple aspects of neuro-cognition and psychophysiological responses.

After examining the data, outliers were winsorized to -4 to 4 z-scores to avoid skewing a clustering solution. Then, data from participants with psychosis was standardized and submitted to a k-means clustering algorithm. GAP statistics and other clustering validation tools were used to verify that a 3 cluster solution was optimal. This analysis was performed on both B-SNIP1 subjects (n=711) and B-SNIP2 subjects (n=714), with a cross-validation of 88-89% in each sample, indicating a robust replication in independently collected samples.

*Biotypes results*

**Supplementary Table 1.** *Biotype groups demographic and clinical data*

|  | Biotype 1 | Biotype 2 | Biotype 3 | Healthy controls | *p*-value |
| --- | --- | --- | --- | --- | --- |
| Total (n) | 138 | 122 | 133 | 296 |  |
| Age (mean (SD)) | 36.59 (12.39) | 34.37 (12.65) | 32.54 (11.34) | 35.98 (12.25) | 0.020 |
| Sex F (n (%)) | 70 (50.7) | 62 (50.8) | 63 (47.4) | 168 (56.9) | 0.257 |
| Race (n(%)) |  |  |  |  | <.001 |
| AA | 70 (50.7) | 43 (35.2) | 16 (12.0) | 80 (27.1) |  |
| AE | 1 ( 0.7) | 0 ( 0.0) | 0 ( 0.0) | 1 ( 0.3) |  |
| AS | 1 ( 0.7) | 2 ( 1.6) | 3 ( 2.3) | 14 ( 4.7) |  |
| CA | 61 (44.2) | 70 (57.4) | 107 (80.5) | 187 (63.4) |  |
| MR | 3 ( 2.2) | 3 ( 2.5) | 3 ( 2.3) | 6 ( 2.0) |  |
| NH | 0 ( 0.0) | 0 ( 0.0) | 0 ( 0.0) | 1 ( 0.3) |  |
| OT | 2 ( 1.4) | 4 ( 3.3) | 4 ( 3.0) | 6 ( 2.0) |  |
| Site (n(%)) |  |  |  |  | <.001 |
| Dallas | 17 (12.3) | 14 (11.5) | 16 (12.0) | 47 (15.9) |  |
| Hartford | 25 (18.1) | 28 (23.0) | 22 (16.5) | 57 (19.3) |  |
| Baltimore | 53 (38.4) | 35 (28.7) | 25 (18.8) | 51 (17.2) |  |
| Chicago | 25 (18.1) | 24 (19.7) | 47 (35.3) | 61 (20.6) |  |
| Detroit | 2 ( 1.4) | 5 ( 4.1) | 7 ( 5.3) | 40 (13.5) |  |
| Boston | 16 (11.6) | 16 (13.1) | 16 (12.0) | 40 (13.5) |  |
| CPZ (mean (SD)) | 473.08 (367.63) | 500.56 (436.24) | 409.88 (417.31) | N/A | 0.341 |
| Duration of illness (mean (SD)) | 18.56 (12.25) | 16.07 (11.02) | 16.21 (11.18) | N/A | 0.146 |
| PANSS (mean (SD)) | 62.33 (16.82) | 64.83 (17.07) | 59.86 (17.05) | N/A | 0.073 |
| BACS (mean (SD)) | -1.90 (1.27) | -1.82 (1.27) | -0.39 (1.21) | 0.02 (1.20) | <0.001 |

*Note.* Information is missing for the following variables: sex (n=1 control); race (n=1 control); duration of illness (n=10 patients); PANSS (n=9 patients); CPZ (n=136 patients); BACS (n=18 controls and n=7 patients); SD, Standard deviation; F, female; CA, Caucasian; AA, African American; AE, American Indian; AS, Asian; MR, Mixed Race; NH, Native Hawaiian; OT, Other; BACS, Brief Assessment of Cognition in Schizophrenia Total Composite Z score; PANSS, Positive and Negative Syndrome Scale Total; CPZ, chlorpromazine equivalents.

***
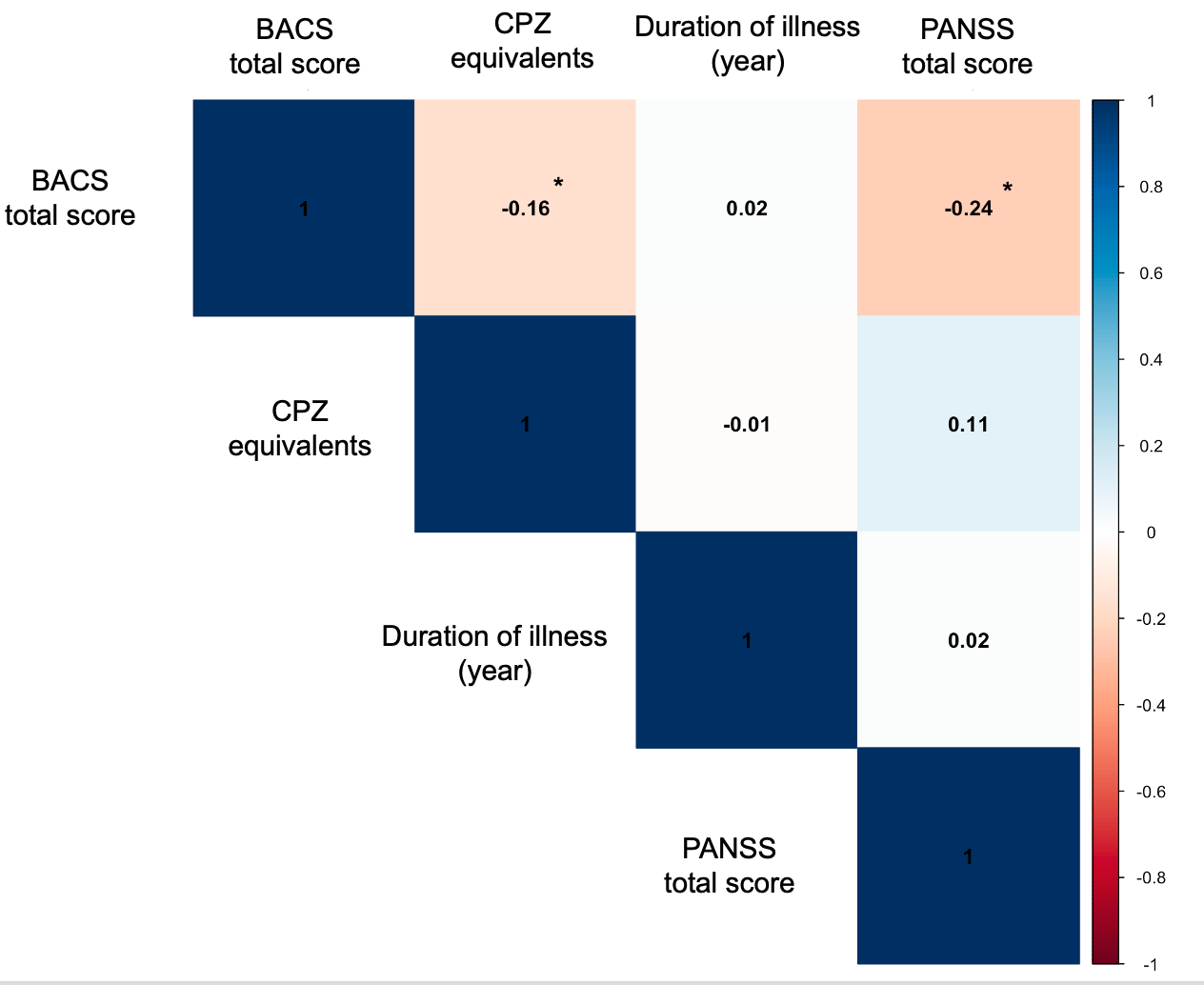
***

**Supplementary Fig 3.** Pearsons Correlations between each pair of variables. BACS, Brief Assessment of Cognition in Schizophrenia Total Composite Z score; PANSS, Positive and Negative Syndrome Scale Total; CPZ, chlorpromazine equivalents.

*Supplementary references*

1. Clementz, B. A., Parker, D. A., Trotti, R. L., McDowell, J. E., Keedy, S. K., Keshavan, M. S., Pearlson, G. D., Gershon, E. S., Ivleva, E. I., Huang, L.-Y., Hill, S. K., Sweeney, J. A., Thomas, O., Hudgens-Haney, M., Gibbons, R. D., & Tamminga, C. A. (2022). Psychosis Biotypes: Replication and Validation from the B-SNIP Consortium. Schizophrenia Bulletin, 48(1), 56–68. https://doi.org/10.1093/schbul/sbab090

2. Clementz, B. A., Sweeney, J. A., Hamm, J. P., Ivleva, E. I., Ethridge, L. E., Pearlson, G. D., Keshavan, M. S., & Tamminga, C. A. (2016). Identification of distinct psychosis biotypes using brain-based biomarkers. American Journal of Psychiatry, 173(4), 373–384. https://doi.org/10.1176/appi.ajp.2015.14091200

3. Ethridge, L. E., Ph, D., Hamm, J. P., Pearlson, G. D., Carol, A., Sweeney, J. A., Ph, D., Keshavan, M. S., Brett, A., Tamminga, C. A., Sweeney, J. A., Keshavan, M. S., Clementz, B. A., Ph, D., Hamm, J. P., Pearlson, G. D., Carol, A., Sweeney, J. A., Ph, D., … Brett, A. (2017). Event-Related Potential and Time-Frequency Endophenotypes for Schizophrenia and Psychotic Bipolar Disorder. Biological Psychiatry, 77(2), 127–136.

4. Ethridge, L. E., Soilleux, M., Nakonezny, P. A., Reilly, J. L., Kristian Hill, S., Keefe, R. S. E., Gershon, E. S., Pearlson, G. D., Tamminga, C. A., Keshavan, M. S., & Sweeney, J. A. (2014). Behavioral response inhibition in psychotic disorders: Diagnostic specificity, familiality and relation to generalized cognitive deficit. Schizophrenia Research, 159(2–3), 491–498. https://doi.org/10.1016/j.schres.2014.08.025

5. Gotra, M. Y., Hill, S. K., Gershon, E. S., Tamminga, C. A., Ivleva, E. I., Pearlson, G. D., Keshavan, M. S., Clementz, B. A., McDowell, J. E., Buckley, P. F., Sweeney, J. A., & Keedy, S. K. (2020). Distinguishing patterns of impairment on inhibitory control and general cognitive ability among bipolar with and without psychosis, schizophrenia, and schizoaffective disorder. Schizophrenia Research, 223, 148–157. https://doi.org/10.1016/j.schres.2020.06.033

6. Hamm, J. P., Ethridge, L. E., Boutros, N. N., Keshavan, M. S., Sweeney, J. A., Pearlson, G. D., Tamminga, C. A., & Clementz, B. A. (2014). Diagnostic specificity and familiality of early versus late evoked potentials to auditory paired stimuli across the schizophrenia-bipolar psychosis spectrum. Psychophysiology, 51(4), 348–357. https://doi.org/10.1111/psyp.12185

7. Hill, S.K., Reilly, J. L., Keefe, R. S. E., Gold, J. M., Bishop, J. R., Gershon, E. S., … Sweeney, J. A. (2013). Neuropsychological impairments in schizophrenia and psychotic Bipolar disorder: Findings from the Bipolar-Schizophrenia Network on Intermediate Phenotypes (B-SNIP) study. *American Journal of Psychiatry*, *170*(11), 1275–1284.

8. Huang, L. Y., Jackson, B. S., Rodrigue, A. L., Tamminga, C. A., Gershon, E. S., Pearlson, G. D., Keshavan, M. S., Keedy, S. S., Kristian Hill, S., Sweeney, J. A., Clementz, B. A., & McDowell, J. E. (2022). Antisaccade error rates and gap effects in psychosis syndromes from bipolar-schizophrenia network for intermediate phenotypes 2 (B-SNIP2). Psychological Medicine, 52(13), 2692–2701. https://doi.org/10.1017/S003329172000478X

9. Keefe, R. S. E., Goldberg, T. E., Harvey, P. D., Gold, J. M., Poe, M. P., & Coughenour, L. (2004). The Brief Assessment of Cognition in Schizophrenia: Reliability, sensitivity, and comparison with a standard neurocognitive battery. Schizophrenia Research, 68(2–3), 283–297. https://doi.org/10.1016/j.schres.2003.09.011

10. Parker, D. A., Trotti, R. L., McDowell, J. E., Keedy, S. K., Gershon, E. S., Ivleva, E. I., Pearlson, G. D., Keshavan, M. S., Tamminga, C. A., Sweeney, J. A., & Clementz, B. A. (2020). Auditory paired-stimuli responses across the psychosis and bipolar spectrum and their relationship to clinical features. Biomarkers in Neuropsychiatry, 3. https://doi.org/10.1016/j.bionps.2020.100014

11. Parker, D. A., Trotti, R. L., McDowell, J. E., Keedy, S. K., Hill, S. K., Gershon, E. S., Ivleva, E. I., Pearlson, G. D., Keshavan, M. S., Tamminga, C. A., & Clementz, B. A. (2021). Auditory Oddball Responses Across the Schizophrenia-Bipolar Spectrum and Their Relationship to Cognitive and Clinical Features. The American Journal of Psychiatry, 178(10), 952–964. https://doi.org/10.1176/appi.ajp.2021.20071043

12. Reilly, J. L., Frankovich, K., Hill, S., Gershon, E. S., Keefe, R. S. E., Keshavan, M. S., Pearlson, G. D., Tamminga, C. A., & Sweeney, J. A. (2014). Elevated antisaccade error rate as an intermediate phenotype for psychosis across diagnostic categories. Schizophrenia Bulletin, 40(5), 1011–1021. https://doi.org/10.1093/schbul/sbt132
